# COXPRESdb v8: an animal gene coexpression database navigating from a global view to detailed investigations

**DOI:** 10.1093/nar/gkac983

**Published:** 2022-11-09

**Authors:** Takeshi Obayashi, Shun Kodate, Himiko Hibara, Yuki Kagaya, Kengo Kinoshita

**Affiliations:** Graduate School of Information Sciences, Tohoku University, 6-3-09, Aramaki-Aza-Aoba, Aoba-ku, Sendai, 980-8679, Japan; Tohoku Medical Megabank Organization, Tohoku University, Sendai, 980-8573, Japan; Graduate School of Information Sciences, Tohoku University, 6-3-09, Aramaki-Aza-Aoba, Aoba-ku, Sendai, 980-8679, Japan; Department of Biological Sciences, Purdue University, West Lafayette, IN 47907, USA; Graduate School of Information Sciences, Tohoku University, 6-3-09, Aramaki-Aza-Aoba, Aoba-ku, Sendai, 980-8679, Japan; Tohoku Medical Megabank Organization, Tohoku University, Sendai, 980-8573, Japan; Institute of Development, Aging, and Cancer, Tohoku University, Sendai, 980-8575, Japan

## Abstract

Gene coexpression is synchronization of gene expression across many cellular and environmental conditions and is widely used to infer the biological function of genes. Gene coexpression information is complex, comprising a complete graph of all genes in the genome, and requires appropriate visualization and analysis tools. Since its initial release in 2007, the animal gene expression database COXPRESdb (https://coxpresdb.jp) has been continuously improved by adding new gene coexpression data and analysis tools. Here, we report COXPRESdb version 8, which has been enhanced with new features for an overview, summary, and individual examination of coexpression relationships: CoexMap to display coexpression on a genome scale, pathway enrichment analysis to summarize the function of coexpressed genes, and CoexPub to bridges coexpression and existing knowledge. COXPRESdb also facilitates downstream analyses such as interspecies comparisons by integrating RNAseq and microarray coexpression data in a union-type gene coexpression. COXPRESdb strongly support users with the new coexpression data and enhanced functionality.

## INTRODUCTION

Living systems, from cells to individuals to populations, have a complex hierarchical structure, and the coordination of genes is fundamental to construct and maintain this system. Gene coexpression is synchronization of gene expression across many cellular and environmental conditions and is widely used to infer the biological function of genes (1–3). Since a larger number of samples improves the quality of coexpression information (4,5), many coexpression databases have been developed based on a meta-analysis of publicly available gene expression data (6–11). Although the idea of gene coexpression is simple, the actual calculation involves many technical and conceptual issues, including sample selection, normalization within and across experiments, and coexpression indices. Many studies, including benchmark studies, have been performed on this subject (4,5,8,12–17).

One of the most natural ways to represent the coexpression information is a gene list ordered by coexpression strength for a given guide gene (1). Database users can simply examine the coexpressed genes one by one from the top of the list to search for functionally related genes to the guide gene. On the other hand, gene relationship is not as simple as to represent on list. The individual genes on the list also have coexpression relationships with each other. Gene network represents such a many-gene relationship. However, coexpression networks are a kind of correlation network and tend to be dense networks with high clustering coefficients, sometimes colloquially referred to as hairballs. In addition, coexpression value is assigned for every gene pair, meaning that coexpression network is a weighted complete graph. To effectively show coexpression information as a network, binarization of the display and non-display of edges is necessary, resulting in loss of information.

Due to the difficulty of simply understanding gene coexpression information, various analyses have been proposed. As a macroscopic analysis, it is possible to display all genes by ignoring the individual relationships of gene pairs and placing nodes without edges (18). For pathway-level summarization, enrichment analysis of functional annotation of coexpressed genes is helpful (11,19). After an overview of gene coexpression relationships, an individual examination is necessary. Some databases such as STRING-DB incorporates multiple data sources including a text mining of scientific publications (11).

We have developed COXPRESdb, a gene coexpression database for animals. COXPRESdb has been continuously improved with new coexpression data and analysis tools since it was first released for human and mouse in 2007 (20–24). COXPRESdb provides gene lists and gene networks as basic functionalities. The coexpressed gene list is displayed as a parallel view of different species and platforms for comparison. The COXPRESdb gene network uses a set of the top three coexpression edges from all genes in the genome, based on the same idea as transitive reduction, which draws only A-B and B-C and omits the presumed A-C. This drawing rule improves the visibility of the network structure, but the local network around a gene of interest remains a dense network. Therefore, the coexpressed gene network in the gene page uses only the 20 genes that are directly or indirectly coexpressed with the guide gene. COXPRESdb version 8 offers enhanced capabilities with new features for an overview, summary, and individual examination of coexpression relationships: CoexMap displays coexpression on a genome scale; pathway enrichment analysis summarizes the functions of the coexpressed gene list; and CoexPub bridges coexpression and existing knowledge. In addition, a union-type coexpression, which integrates RNAseq and microarray coexpression data, facilitates downstream analyses such as interspecies comparisons. COXPRESdb has been enhanced to assist in exploring complex gene networks for molecular biological studies.

## OVERVIEW OF THE LATEST COEXPRESSION DATA

### Global similarity among coexpression platforms in COXPRESdb ver. 8.1

Since the last report for COXPRESdb version 7.0 (24), we have updated COXPRESdb with one major version and four minor versions. In addition to the update of all the pre-existing coexpression data, we have added the cat coexpression data since version 8.1 (Table 1). Cats have had a close relationship with humans and are an important model species for medical and veterinary research (25–28). As per our strategy, COXPRESdb independently calculates RNAseq-based and microarray-based coexpression values and then compares the two to examine the reliability of the coexpression information (24). However, it is not convenient to always use multiple coexpression data for downstream analyses, including interspecies comparison. Since version 7.1, we have provided a union-type coexpression data for each species, which is the average of RNAseq-based and microarray-based coexpression values. For gene pairs that do not have microarray data, we use RNAseq coexpression values with a shrinkage penalty.

**Table 1.** Coexpression data in COXPRESdb version 8.1

Species	Version	Release date	Samples	KEGG score	GO score
**Nematode**	Cel-u.c3-1	2022.06.30		7.250	5.508
**Nematode**	Cel-m.c5-0	2021.12.16	1357	5.966	4.552
**Nematode**	Cel-r.c3-0	2021.12.16	5785	7.304	5.468
**Dog**	Cfa-u.c3-1	2022.06.30		4.577	1.459
**Dog**	Cfa-m.c4-0	2021.12.16	619	3.364	1.065
**Dog**	Cfa-r.c3-0	2021.12.16	1361	4.019	1.380
**Fly**	Dme-u.c3-1	2022.06.30		7.050	4.544
**Fly**	Dme-m.c5-0	2021.12.16	3401	6.394	4.105
**Fly**	Dme-r.c4-0	2021.12.16	13514	6.659	4.326
**Zebrafish**	Dre-u.c3-1	2022.06.30		7.835	5.254
**Zebrafish**	Dre-m.c5-0	2021.12.16	1321	9.020	6.264
**Zebrafish**	Dre-r.c3-0	2021.12.16	10037	7.777	5.192
**Domestic cat**	Fca-r.c1-0	2022.06.30	267	3.610	
**Chicken**	Gga-u.c3-1	2022.06.30		5.236	2.282
**Chicken**	Gga-m.c5-0	2021.12.16	1155	3.283	1.794
**Chicken**	Gga-r.c3-0	2021.12.16	3333	5.558	2.248
**Human**	Hsa-u.c4-0	2022.06.30		6.302	2.995
**Human**	Hsa-m.c7-0	2021.12.16	25362	4.343	2.182
**Human**	Hsa-m2.c4-0	2021.12.16	10511	4.860	2.493
**Human**	Hsa-r.c6-0	2022.06.30	235187	6.105	2.800
**Monkey**	Mcc-u.c3-1	2022.06.30		4.301	
**Monkey**	Mcc-m.c4-0	2021.12.16	590	2.200	
**Monkey**	Mcc-r.c3-0	2021.12.16	5665	4.321	
**Mouse**	Mmu-u.c4-0	2022.06.30		7.205	3.335
**Mouse**	Mmu-m.c5-0	2021.12.16	25087	6.220	2.955
**Mouse**	Mmu-r.c6-0	2022.06.30	214753	6.953	3.150
**Rat**	Rno-u.c3-1	2022.06.30		6.977	2.745
**Rat**	Rno-m.c5-0	2021.12.16	7872	6.735	2.383
**Rat**	Rno-r.c3-0	2021.12.16	13267	6.329	2.519
**Budding yeast**	Sce-u.c3-1	2022.06.30		9.143	4.712
**Budding yeast**	Sce-m.c4-0	2021.12.16	3071	9.347	4.398
**Budding yeast**	Sce-r.c3-0	2021.12.16	6225	8.791	4.509
**Fission yeast**	Spo-u.c3-1	2022.06.30		5.782	3.222
**Fission yeast**	Spo-m.c4-0	2021.12.16	166	3.539	2.190
**Fission yeast**	Spo-r.c3-0	2021.12.16	556	5.892	3.061

**Figure 1.**
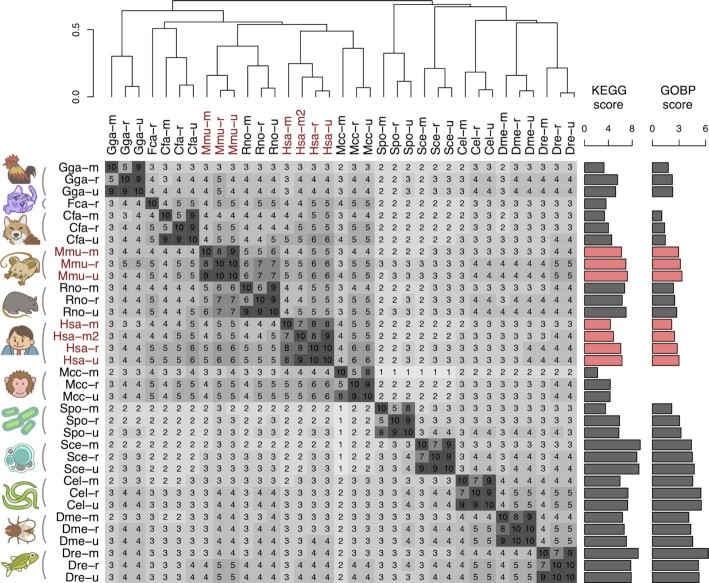
Similarity among the coexpression platforms in COXPRESdb version 8.1. The Pearson correlation coefficients of the coexpression z-scores among the 35 coexpression platforms are shown in each cell as a 10-fold rounded value, which is also indicated by shading. The coexpression platforms are hierarchically clustered by the average linkage method. KEGG and GOBP scores indicate a degree of consistency of the gene coexpression data with the gene annotations. The GOBP annotations were not available for cat (Fca) and macaque (Mcc), so the GOBP scores for these two species are blank. The human and mouse platforms are the most heavily used in COXPRESdb and are highlighted in red.

Similarities among all coexpression platforms in COXPRESdb ver 8.1 are summarized in Figure 1. We performed this comparison using one-to-one orthologous genes in the 12 species. Based on the ortholog calculation in COXPRESdb using OrthoFinder (29), there were 656 one-to-one orthologous genes, composing 214 840 gene pairs in each species. The Pearson correlation coefficients using the 214 840 gene pairs among the 35 coexpression platforms are shown in each cell as a 10-fold rounded value (Figure 1). The coexpression platforms are hierarchically clustered by the average linkage method, revealing that interspecies coexpression similarity reflects evolutionary relationships among species, as we reported previously (24). The new cat RNAseq coexpression (Fca-r) is closest to the canine coexpression, as expected (Figure 1).

In each species, the union-type gene coexpression data is more similar to the RNAseq coexpression data than the microarray coexpression data. This phenomenon is primarily due to two factors. First, about 40% of all gene pairs do not have microarray coexpression values (Supplementary Table S1). In this case, union-type gene expression only uses RNAseq coexpression values with a penalty, resulting in similar coexpression values between the union and RNAseq coexpression data. Second, RNAseq-based coexpression tends to show larger variance of coexpression values for the more highly expressed genes (Supplementary Figure S1), as reported (30). In contrast, this trend was less pronounced for microarray coexpression (Supplementary Figure S2). One-to-one orthologous genes among the 12 species, including yeast, are expected to be highly expressed because of their housekeeping functions. As a result, RNAseq gene coexpression values of one-to-one ortholog gene pairs predominantly take over the union-type coexpression data. CoexMap, described below, successfully visualizes the characteristics of the one-to-one orthologous gene pairs.

### Quality assessment of coexpression data by pathway annotations

To assess the quality of individual gene coexpression data, we quantified the consistency between the gene coexpression and the functional annotations of KEGG pathway (31) and Gene Ontology Biological Process (GOBP) (32), which we have denoted as KEGG and GOBP scores (13,24). Comparison of these scores between the current and previous versions revealed the gradual improvement of these scores (Supplementary Figures S3 and S4). The union-type coexpression, which is the default platform in the tools in COXPRESdb, stably scored higher than RNAseq and microarray gene coexpression data, supporting the suitability of the union coexpression data as representative of a species. The scores for the current version (8.1) are also shown on the right side of the similarity matrix in Figure 1. In mammalian species, human, mouse, and rat, have relatively better scores, reflecting their enormous amount of gene expression data (Table 1). The lower scores of the microarray coexpression for macaque monkey (Mcc-m) and chicken (Gga-m) were consistent with lower correlation of these platforms with the others (Figure 1).

## NEW FUNCTIONALITIES

### Coexpressed gene list

**Figure 2.**
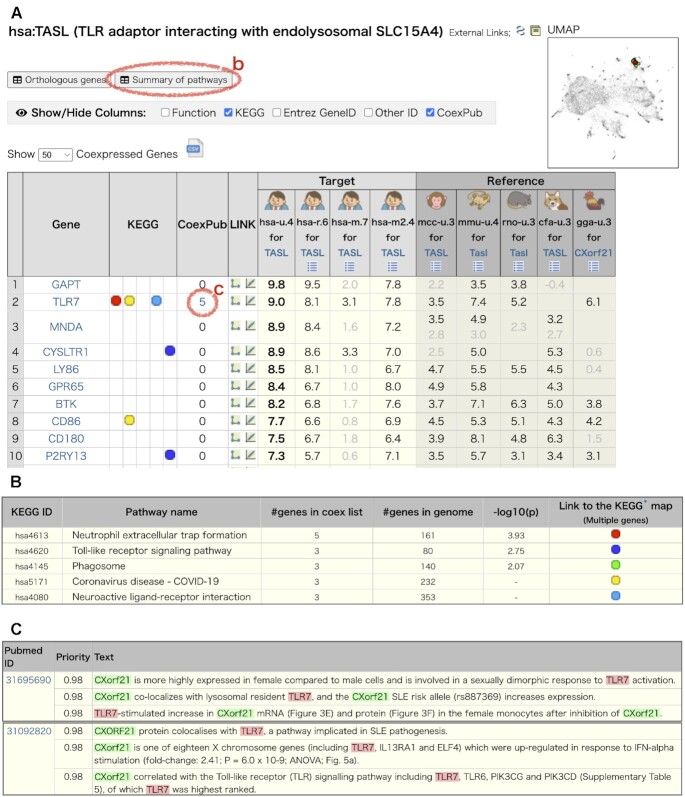
The coexpressed gene list page for the human *TASL* gene. (**A**) The coexpressed genes are listed in descending order of coexpression with the query gene, *TASL* (*CXorf21*). https://coxpresdb.jp/gene_coexpression/?gene_id=80231. (**B**) Summary of KEGG pathways in the coexpressed gene list. (**C**) The CoexPub page for *TASL* and *TLR7*, which is linked from the CoexPub column in (A).

The coexpressed gene list provides a direct approach to investigating gene coexpression information. This page has been enhanced with new functionalities since our previous report for version 7.0 (24). For demonstration, we focus on the *CXorf21* (*TASL*) gene, which is one of the causative candidate genes in a GWAS study for human autoimmune disease, Systemic lupus erythematosus (33,34). Odhams et al. reported coexpression between *CXorf21* and genes for Toll-like receptor (TLR) signalling pathway using the Hsa-r.c1-0 coexpression data in COXPRESdb version 6.0 and then experimentally determined the colocalization of CXorf21 with TLR7 in B cells by a structured illumination microscopy technique (34). Here, we show how the current COXPRESdb (version 8.1) supports this study. Figure 2A is the coexpressed gene list page for *TASL* (*CXorf21*). The summary of the KEGG pathway enrichment analysis helps understand the coexpressed gene list as a whole, shown by clicking on ‘summary of pathways’, displaying that the top-50 gene list includes the Toll-like receptor signaling pathway (KEGG pathway: hsa04620) (Figure 2B), as reported (34). The coexpressed genes are ordered according to the union-type human coexpression data (Hsa-u.c4-0), which is indicated by bold coexpression values in the 6th column of the table. The most strongly coexpressed genes are *GAPT* (GRB2 binding adaptor protein, transmembrane) and *TLR7*, with coexpression z-scores of 9.8 and 9.0, respectively. In COXPRESdb, the coexpression z-scores follow an almost perfectly normal distribution except for those above three (Supplementary Figure S5). Given that the coexpression z-scores from random expression profiles are normally distributed, a coexpression z-score of three is a possible threshold of coexpression reflecting actual co-regulation in a cell. Compared with this threshold, the coexpression z-scores for *TASL* are remarkably high. In particular, the strong coexpression between *TASL* and *TLR7* is consistent with their colocalization (34). To highlight strong coexpression, z-scores less than 3 are shown in a lighter color in the coexpressed gene list (Figure 2A).

A reliability of the union-type coexpression can be checked by individual platforms in the same species (Hsa-r.c6, Hsa-m.c7, Hsa-m2.c4). The union-type gene coexpression (Hsa-u) is the z-score of the average of the RNAseq (Hsa-r) and microarray (Hsa-m2) gene coexpression values. In this coexpressed gene list, these two types of gene coexpression data are consistent. The other microarray platform, Hsa-m, which was not used to calculate union-type gene coexpression, has a much weaker gene coexpression but still shows a similar coexpression trend. On the right side of the list, union-type coexpression for other species is displayed as a cross-species reference to assess the importance of the coexpression (21). Almost all gene coexpression with the human *TASL* gene is also observed in macaque, mouse, rat, dog, and chicken, indicating its stability in evolution.

A survey of relevant scientific reports is crucial to further examine a coexpressed gene of interest. To support this step, we develop a new tool, CoexPub, by machine learning against the gene-publication association data on PubTator Central (35). The CoexPub column in Figure 2A shows that there are five reports describing functional relationships between *TASL* and *TLR7*. By clicking on the number ‘5’, CoexPub displays the most relevant sentences describing the functional relationship between *TASL* (*CXorf21*) and *TLR7* for each of the five papers (Figure 2C), where the second article is by Odhams et al. (34).

### CoexMap

**Figure 3.**
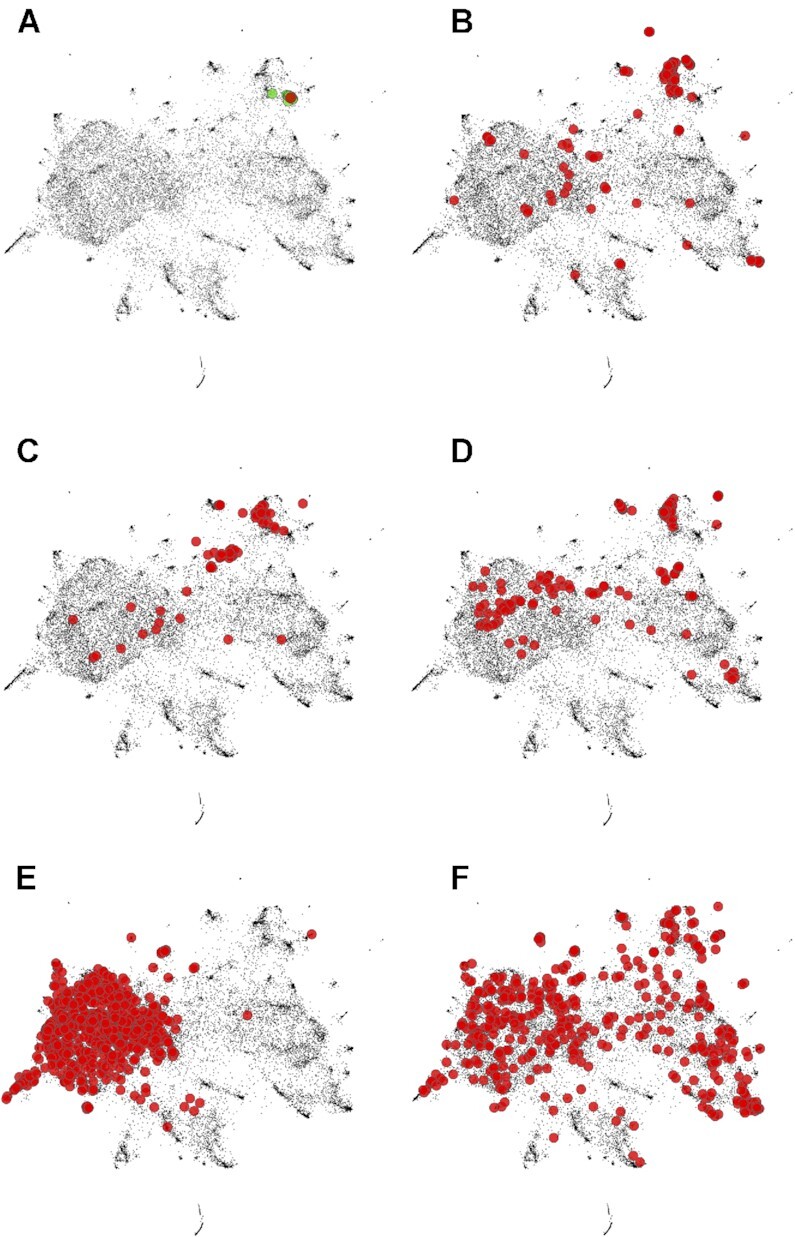
Coexpression map for human gene coexpression. (**A**) *TASL* and its top-20 coexpressed genes. (**B**) The 169 genes in the KEGG hsa04613 pathway (Neutrophil extracellular trap formation). (**C**) The 59 genes in hsa04620 (Toll-like receptor signaling pathway). (**D**) The 145 genes in hsa04145 (Phagosome). (**E**) The 656 one-to-one orthologous genes in the 12 species. (**F**) 500 randomly selected genes out of 1743 genes used to calculate the KEGG score.

The CoexMap is a new tool that displays the location of a given gene in the gene coexpression space constructed by UMAP (36). The coexpression map shows that *TASL* gene (red) and its top 20 coexpressed genes (green) form a compact modular structure in the upper right corner of the map (Figure 3A). The thumbnail of this map is also shown in the upper right corner of the coexpressed gene list page (Figure 2A). Figure 3B–D shows the map for the genes for the three KEGG pathways enriched in Figure 2B. Although the distributions of these pathway genes are various, these pathways commonly occupy the upper right region of the coexpression map, suggesting that a core module of the immune system is located in this region. *TASL* and its coexpressed genes (Figure 3A) are located adjacent to this core region, suggesting a strong association of the *TASL* gene to the core module of immune system.

The coexpression map can visualize global tendency of the one-to-one orthologous genes used in Figure 1. We discussed that one-to-one orthologous genes tend to have a housekeeping function and thus have somewhat different characteristics than randomly selected genes. This idea is clearly visualized in the coexpression map, which shows that the one-to-one orthologous genes entirely cover the dense structure on the left side of the map (Figure 3E). The non-random nature of one-to-one orthologous genes illustrates the difficulty of comparing gene coexpression across distantly related species in an unbiased manner. Similarly, functional annotations are not randomly associated with genes. Highly expressed genes are well studied and, therefore, well-annotated (13). We used 1743 genes associated with informative KEGG terms to calculate of the KEGG scores for Hsa-r and Hsa-u. CoexMap showed a broad distribution of these genes (Figure 3F), suggesting that the KEGG score, while not yet a random selection of genes, provides a more genome-scale assessment.

Note that since the original gene coexpression values are non-Euclidean high-dimensional data, significant information loss due to dimensionality reduction is inevitable. Nevertheless, genome-scale visualization can reveal significant trends in a set of genes. The examples for the immune system and housekeeping functions characterized the overall structure of the coexpression map, with housekeeping genes creating a large structure on the left side and modules of tissue-specific function distributed in the other regions (Figure 3). To analyze multi-layered biological systems, a multi-layered approach is necessary. A suite of functions in COXPRESdb supports multi-layered analysis with high-quality gene coexpression information: CoexMap for genome-scale viewing, NetworkDrawer for a selected gene set, coexpressed genes list for a guide gene, and CoexPub for a gene pair. COXPRESdb version 8 powerfully supports individual studies in molecular biology with the enhancements of the tools and new coexpression data.

## MATERIALS AND METHODS

### Calculation of coexpression data

The quantification of gene expression data was performed, as reported previously (24). Briefly, Illumina RNAseq data were downloaded from DDBJ Sequence Read Archive (37) and quantified using Matataki (38). After eliminating runs comprising <2M read counts, genes with average counts of <30 are deleted. All read counts are converted to the base-2 logarithms after adding a pseudo-count of 0.125. Affymetrix microarray data were downloaded from EBI ArrayExpress (39) and quantified by the RMA method (40). A batch correction was performed using Combat (41), where the SRP units and the download units were used as the batch units. The expression matrix was rearranged by row-centered principal component analysis, and the top 1000 principal components were used as the re-organized samples.

The main part of coexpression calculation methodology was performed as previously described (14,24). In each iteration of Subsampling Aggregating (Subagging) of the gene-to-gene correlation calculation, we subsampled 100 (ver. 7.0) or 50 (after ver. 7.1) principal components from the 1000 principal components and calculated Mutual Rank (MR) of Pearson correlation coefficient (PCC) for each gene pair (12). After a logit transformation of the MR values (13), the 1000-times iterative calculations were integrated by average (14). Since version 8.0, the final coexpression values are standardized to z-scores in each platform to easily compare the coexpression values among different platforms and versions (Supplementary Figure S5).

In COXPRESdb version 8.1, the numbers of RNAseq runs for human and mouse are enormous, exceeding 200 000 each (Table 1). To handle these massive amounts of RNAseq data, we partitioned the data into seven subsets of experiments for each species and performed the same coexpression calculation procedure described above for each subset. We decided the number of subsets as small as possible because the total computation time will be nearly proportional of the subset number. The genes in each of the seven coexpression datasets differed due to the gene filtering step. We selected genes included in more than three of the seven coexpression data so that all gene pairs in the integrated coexpression data will have gene coexpression values in at least one subgroup. The multiple coexpression values for a gene pair were integrated by average with a penalty in terms of data coverage as follows,

$$\begin{equation*}{\left( {\frac{m}{n}} \right)^k}\frac{1}{m}\sum {z_i},\end{equation*}$$

where ${z_i}$ is coexpression z-score in the *i*-th subset, *n* is the total number of subsets (7 in this case), *m* is the number of subsets including the gene pair of interest (from 1 to 7 in this case), and *k* is a parameter to determine the strength of shrinkage for low reliability penalty. Since the optimal value of *k* varies between 0.1 and 1 for different species based on the KEGG score (data not shown), we commonly used *k* = 0.2 for all species in version 8.1.

The union-type coexpression is the average of RNAseq and microarray coexpression z-scores. For gene pairs with only RNAseq coexpression available, we used the RNAseq coexpression value with a shrinkage. It was done by linear regression (14) in versions 7.1, 7.2 and 8.0 and using the same shrinkage formula above with *n* = 2, *m* = 1 and *k* = 0.2 in version 8.1.

### Evaluation of coexpression

We used the same evaluation protocol reported previously (14). We downloaded the GOBP annotation (32), the KEGG pathway annotation and KEGG Ortholog data (31) on 2022-01-20, 2021-08-17 and 2020-05-26, respectively. For GOBP annotations, we first mapped gene association information on the children's terms to all parents' terms. Then, we selected highly informative terms associated with <50 genes. Using these gene annotations, all gene pairs were divided into groups with and without shared annotation terms. The consistency between gene coexpression and sharing functional annotation was assessed by ROC curves for moving thresholds of coexpression values. As the evaluation index, the partial area under the ROC curve with a false positive rate between 0 and 0.01 was used after being scaled by a factor of 10 000 so that 0.5 indicates a random prediction. Note that gene pairs in the same orthologous group were excluded from the evaluation to reduce the effect of large gene families (14).

### CoexMap

CoexMap is a new tool that displays gene coexpression relationships on a genome-scale. A two-dimensional map of all genes with coexpression information was created using the UMAP algorithm (36) via the ‘uwot’ package in R with a parameter, n_neighbors = 10. As the distance matrix, we used the negative values of the coexpression z-scores. For efficient visualization, points (genes) that are extremely far from the center of the map are moderately displaced.

### CoexPub

CoexPub links coexpressed gene pairs to existing knowledge in scientific papers. Since many gene names appear in scientific papers, their functional implications vary from the main topic to methodological appendices. Therefore, we use machine learning to prioritize informative sentences about the functional relationship of a gene pair from gene-publication association data in PubTator Central (35). We manually selected 300 positive sentences describing a functional relationship of human coexpressed genes and the same number of negative sentences. 250 of 300 sentences were used for training and the rest for test. We used a SciBERT pre-trained model (allenai/scibert_scivocab_uncased) (42) from Hugging Face's Transformers library (43) for fine tuning of the binary classification of the positive and negative sentences (learning_rate = 1e-05, epochs = 3). In this learning, the target coexpressed gene names in each sentence were masked as GENEAAA and GENEBBB and set as special tokens (tokenizer: padding = False, truncation = True, max_length = 511). Using the resultant fine-tuned model, we classified the test data, composing 50 positive and negative sentences each, resulting in an accuracy of 0.85 with 46 true positives, 10 false positives, 4 false negatives and 39 true negatives. We applied this model to all sentences including a coexpressed gene pair in human and mouse and presents 754 534 and 510 775 positive sentences in CoexPub, respectively. We assigned a priority of 1.0 to the 300 positive sentences in the training data, meaning that the sentences shown in Figure 2C are purely the result of machine learning. CoexPub was designed and evaluated primarily for human genes but will be upgraded to apply to other species as evaluations continue.

## DATA AVAILABILITY

The coexpression data provided in COXPRESdb are also available via RDF on https://coxpresdb.jp/sparql and in Zenodo, https://zenodo.org/communities/coxpresdb/. The evaluation program of coexpression data and manually curated sentences for CoexPub are available at https://github.com/takeshiobayashi/coex-function-score and https://doi.org/10.5281/zenodo.7069129, respectively.

## Supplementary Material

gkac983_Supplemental_File
