# Supplementary material for: COXPRESdb v8: an animal gene coexpression database navigating from a global view to detailed investigations
Source: Nucleic Acids Res. 2022 Nov 9;51(D1):D80–7. doi: 10.1093/nar/gkac983 (PMC9825429; doi:10.1093/nar/gkac983)
Supplement: gkac983_Supplemental_File [file gkac983_supplemental_file.pdf]

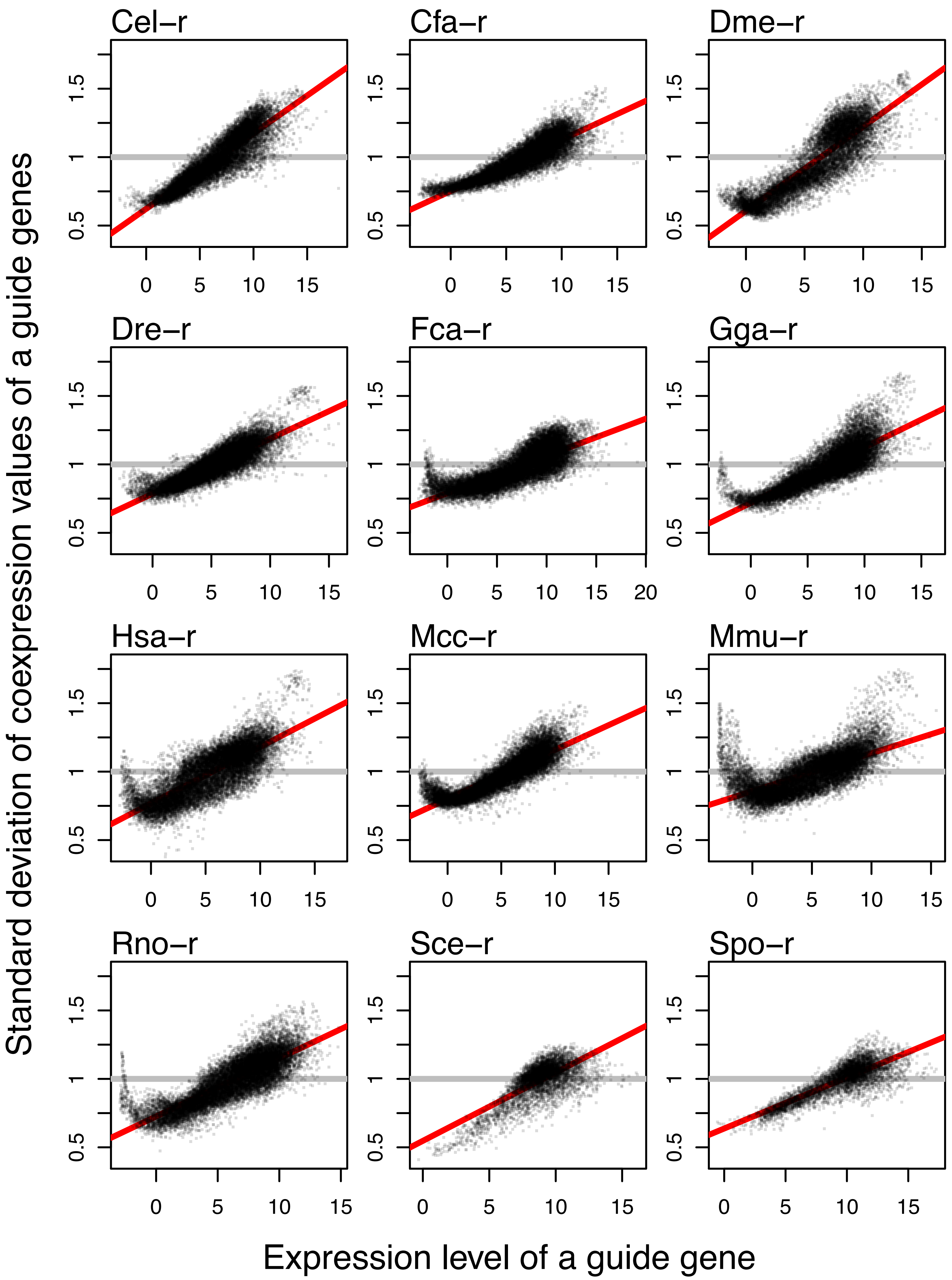

Supplementary Figure S1 Expression level dependency of variation in RNAseq-based gene coexpression values. For every guide gene, the standard deviation of the coexpression z-scores is plotted against the expression level of the gene. The silver line shows the expected standard deviation of the z-scores. The red line is a linear regression of the points showing a trend of expression level dependency.

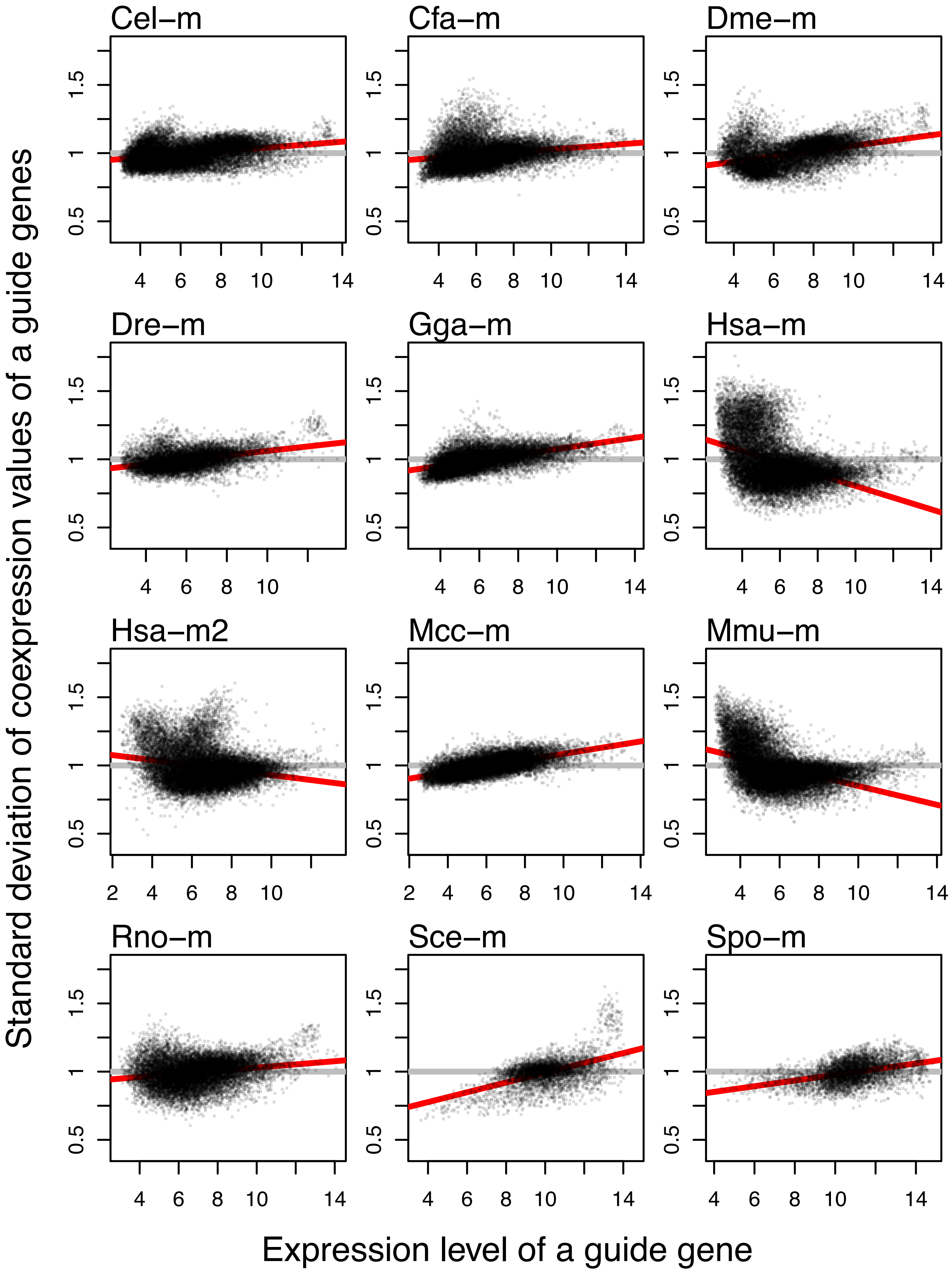

Supplementary Figure S2 Expression level dependency of variation in microarray-based gene coexpression values. Same as Supplementary Figure S1, but for microarray-based coexpression data.

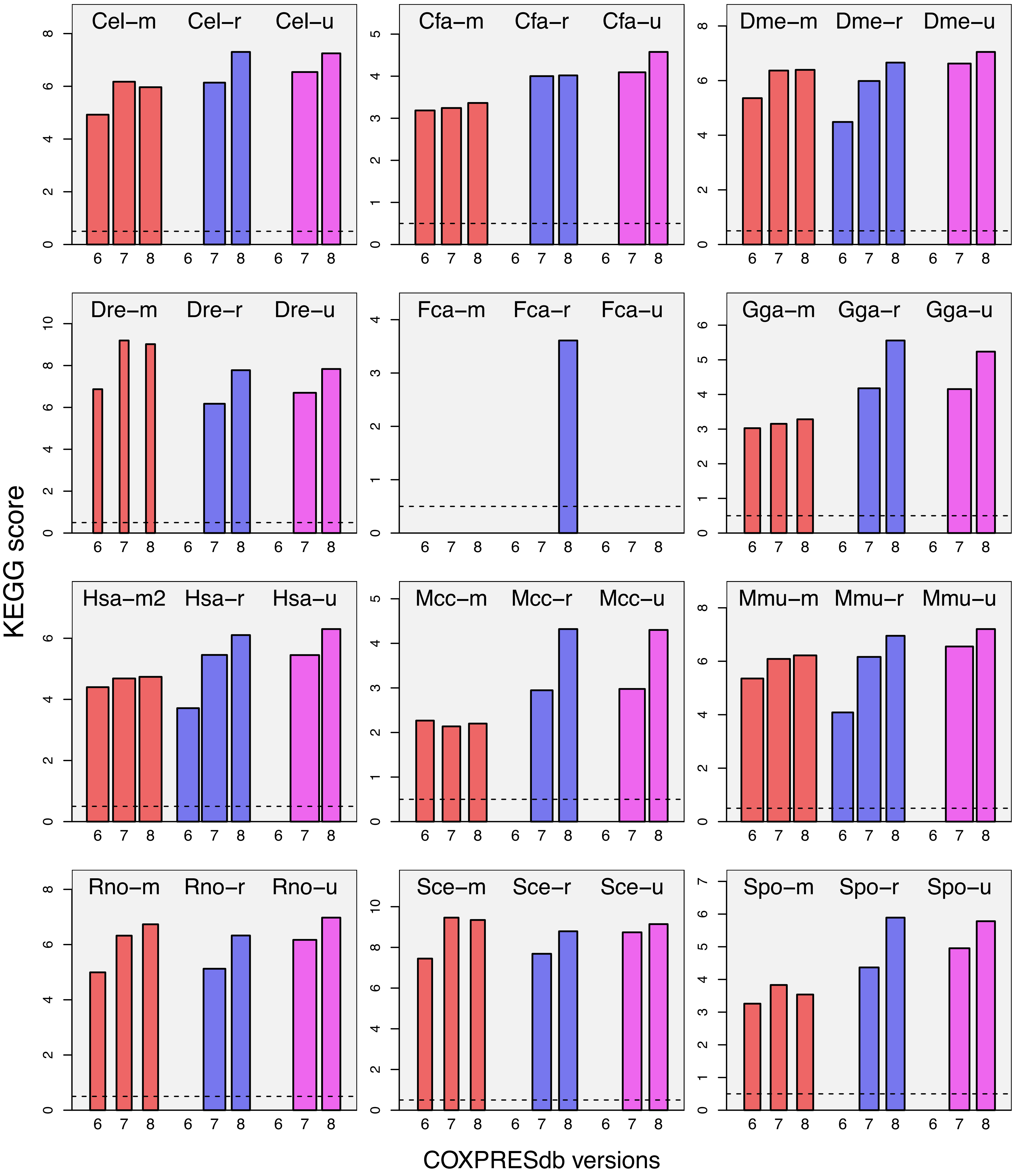

Supplementary Figure S3 Evaluation of coexpression data in COXPRESdb by KEGG pathway annotations. KEGG scores of coexpression data in COXPRESdb versions 6.0, 7.3, and 8.1 are shown. Higher scores indicate better agreement of the coexpression with KEGG pathway annotations. In addition to the microarray-based (Xxx-m) and RNAseq-based (Xxx-r) coexpression data, union-type coexpression (Xxx-u) data are provided since version 7.1. The widths of the bars indicate the gene coverage relative to the number of Xxx-r genes in version 8.1 for each species. The dotted horizontal line of 0.5 indicates the KEGG score for random coexpression data.

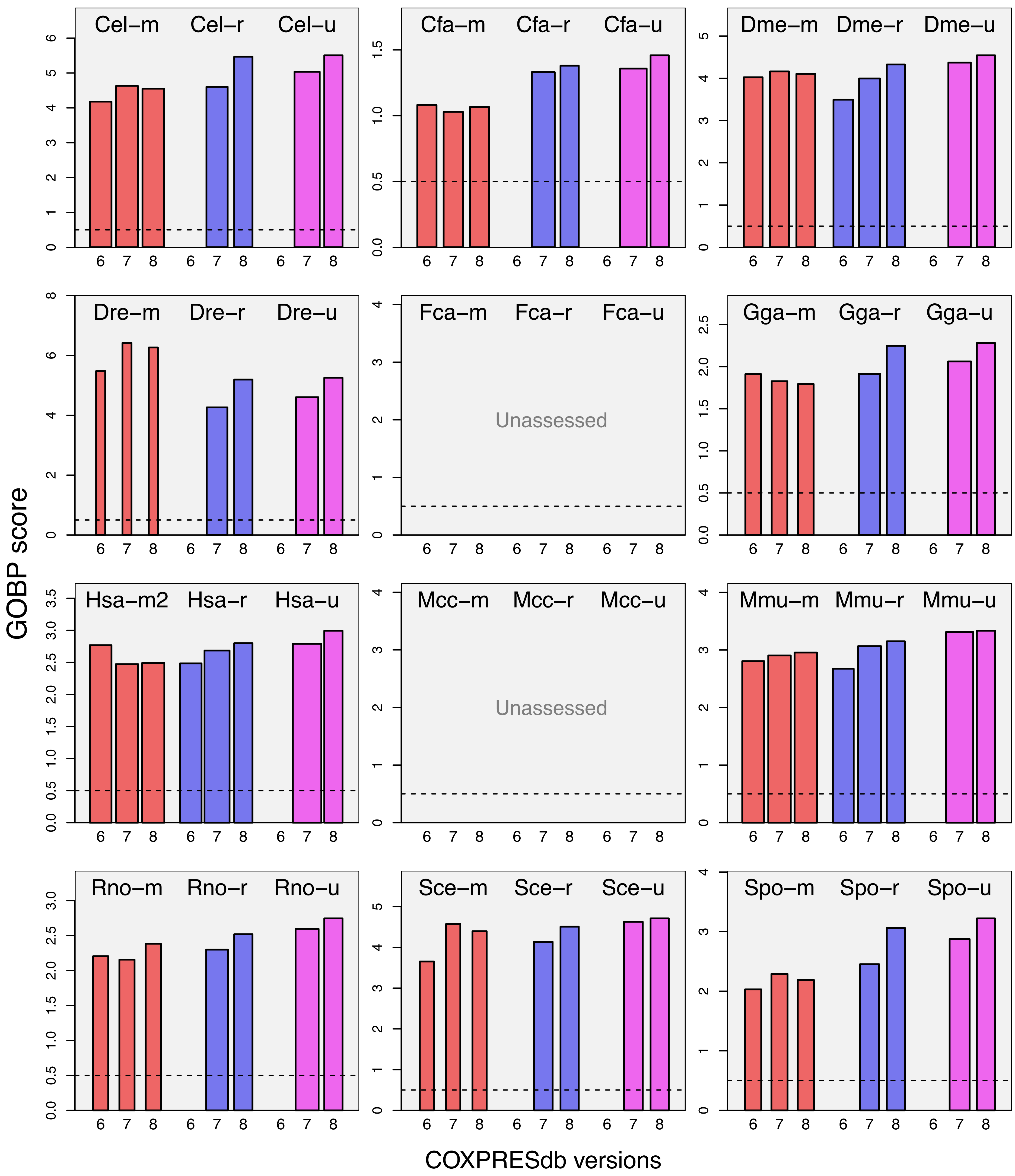

Supplementary Figure S4 Evaluation of coexpression data in COXPRESdb by GOBP annotations. Same as Supplementary Figure S3, but using Gene Ontology Biological Process (GOBP) annotations instead of KEGG pathway annotations.

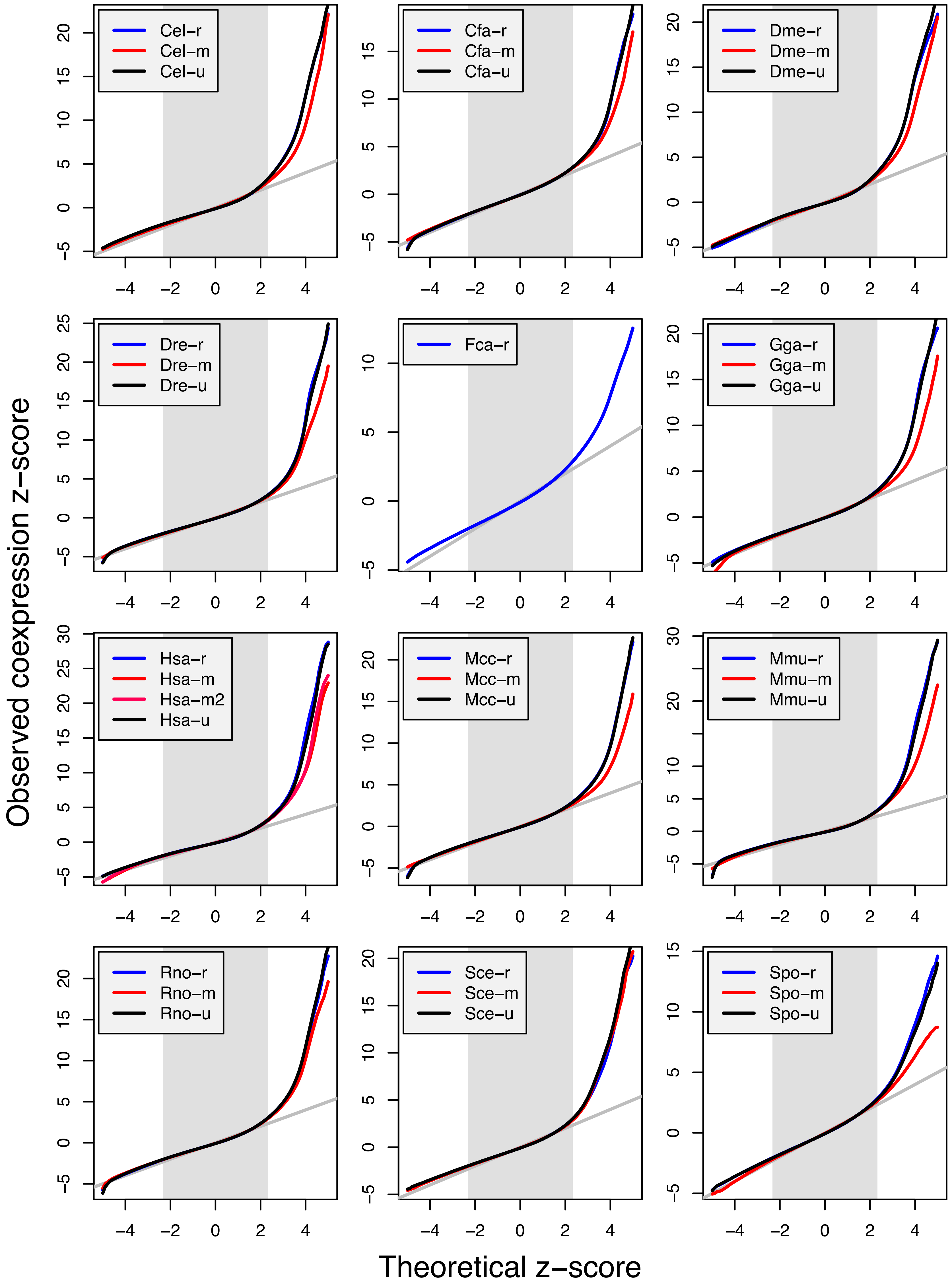

Supplementary Figure S5 QQ plot of coexpression z-score. The horizontal axis is the theoretical z-score with the 1-99 percentile region colored in gray. The vertical axis is the observed coexpression z-score. The top 1% gene coexpression values are significantly greater than the theoretical (random) values.

Supplementary Table S1: RNAseq genes commonly measured in microarray.

Species	Number of RNAseq genes		Proportion in RNAseq gene pairs	
	(a) Total	(b) Common with microarray	(c) Common*	(d) RNAseq-specific
**Cel**	14,532	12,550	75%	25%
**Dme**	12,209	10,866	79%	21%
**Dre**	20,344	8,164	16%	84%
**Gga**	15,998	11,851	55%	45%
**Cfa**	15,365	11,753	59%	41%
**Mmu**	17,008	15,215	80%	20%
**Rno**	16,521	12,267	55%	45%
**Hsa**	16,651	14,896	80%	20%
**Mcc**	16,312	11,198	47%	53%
**Sce**	5,718	4,431	60%	40%
**Spo**	5,512	4,549	68%	32%
